# CondenSimAdapter: A Versatile Builder for Multiscale Simulations of Protein Condensates with Broad Force-Field Compatibility and Robust Dense-Phase Relaxation

**DOI:** 10.1021/acs.jcim.6c00413

**Published:** 2026-06-30

**Authors:** Xiaojing Tian, Wei Han

**Affiliations:** † School of Chemical Biology and Biotechnology, Peking University Shenzhen Graduate School, Shenzhen 518055, China; ‡ Department of Chemistry, Faculty of Science, 26679 Hong Kong Baptist University, Hong Kong, SAR 999077, China; § Institute for Research and Continuing Education (Shenzhen), 26679 Hong Kong Baptist University, Shenzhen 518000, China; ∥ Institute of Chemical Biology, Shenzhen Bay Laboratory, Shenzhen 518132, China

## Abstract

Multiscale molecular dynamics simulations that sequentially couple coarse-grained (CG) sampling with all-atom (AA) simulation are widely used to study biomolecular condensates, yet building such multiscale systems remains a practical challenge. Dense CG condensate configurations must be backmapped and converted into stable, explicitly solvated AA systems—a step where severe steric clashes often prevent production simulation, creating a “relaxation bottleneck”. Here, we introduce **CondenSimAdapter**, a Python package that bridges this resolution gap by integrating SE(3)-transformer–based cg2all backmapping with a physics-inspired optimization protocol (using Gaussian repulsion and soft-core potentials), which succeeds where standard energy minimization fails. **CondenSimAdapter** unifies four CG and nine AA force fields under a single interface. We validated the workflow by (1) demonstrating the robust elimination of major structure conflicts across diverse CG–AA combinations, (2) verifying its functional versatility in preserving the structural integrity of multidomain proteins, and (3) confirming ensemble fidelity via a 2 μs atomistic simulation of a FUS LC condensate that accurately reproduced established macroscopic and microscopic properties. By resolving the dense-phase relaxation bottleneck and providing a highly accessible, streamlined workflow, **CondenSimAdapter** lowers the technical barrier to multiscale condensate simulations and enables systematic, high-throughput studies of protein phase separation. **CondenSimAdapter** is freely available at https://github.com/hanlab-computChem/CondenSimAdapter.

## Graphical abstract

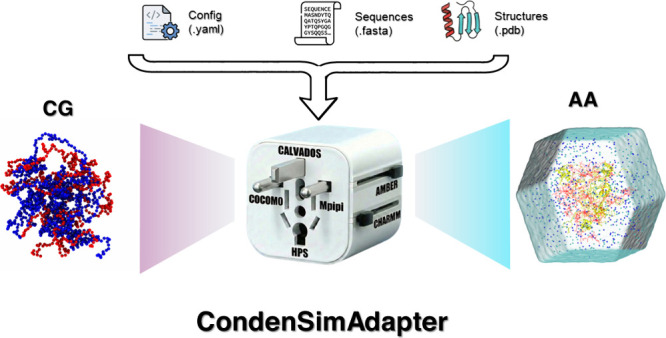

## Introduction

Biological macromolecules can self-assemble into concentrated liquid-like droplets via liquid–liquid phase separation. Commonly referred to as biomolecular condensates or membraneless organelles, these assemblies serve critical functional roles across biological scales, facilitating ribosome assembly,^1^ cellular stress responses,^2^ and modulation of enzymatic reactions.^3^ Uncovering the molecular mechanisms underlying these functions is experimentally challenging due to the dynamic and disordered nature of condensates.^4^ Molecular dynamics simulations have therefore become an important tool for probing the microscopic condensate properties.^5^ Coarse-grained models, especially residue-level models such as HPS,^6^ CALVADOS,^7^ Mpipi,^8^ and COCOMO,^9^ can efficiently sample phase-separated states and sequence-dependent material properties.^10^ In contrast, all-atom (AA) models preserve chemical resolution and are valuable for analyzing side-chain contacts, hydration, ion partitioning, and local dynamics.^11–13^ Direct atomistic equilibration of a protein-rich condensate, however, remains computationally expensive because biologically relevant systems commonly contain many chains and hundreds of thousands of atoms.^5^

To address this initialization bottleneck, sequential multiscale simulation has been developed as a practical strategy.^14–16^ A CG model is first used to generate a dense, pre-equilibrated condensate configuration, which is then converted into an AA representation for explicit-solvent simulation. Existing backmapping approaches, including geometric reconstruction,^17–19^ homology modeling-based rebuilding,^20^ and recent deep-learning coordinate-generation methods,^21–23^ have made it possible to reconstruct atomistic coordinates from reduced representations. While several backmapping approaches have enabled coordinate reconstruction, a critical gap remains: the extreme density of multichain CG configurations often results in severe local steric conflicts that render standard energy minimization ineffective, creating a “relaxation bottleneck” for production simulations.^15,24^

Force-field diversity adds a second practical barrier. CG condensate models can give different physical predictions for the same sequence, even when each model was designed for phase-separating proteins; recent benchmarks show that similar single-chain dimensions do not guarantee similar phase behavior or critical temperatures.^25,26^ AA force fields likewise differ in their balance of folded-protein stability, disordered-chain dimensions, and protein–water interactions.^27–29^ A useful multiscale workflow should therefore lower the practical barrier to constructing and testing alternative CG-to-AA combinations.

To bridge this resolution gap and provide a unified platform for multiscale modeling, we developed **CondenSimAdapter**. Operated via a streamlined terminal CLI, **CondenSimAdapter** integrates four CG force fields, cg2all^21^-based backmapping, a staged Gaussian/soft-core/standard minimization protocol, explicit solvation and ionization, and preparation of simulation-ready systems compatible with GROMACS,^30^ OpenMM,^31^ and Amber.^32^ We then validate **CondenSimAdapter** across three key dimensions: first, its technical robustness in overcoming the relaxation bottleneck across diverse CG force fields; second, its functional versatility in preserving the structural integrity of multidomain proteins across various AA force fields; and third, the ensemble fidelity of its generated configurations, verified through a 2 μs production simulation of a FUS LC condensate.

## CondenSimAdapter Workflow and Tested Systems

**CondenSimAdapter** is organized as a configuration-driven workflow with four stages: initialization, CG sampling, backmapping, and AA minimization (Figure 1). The entire pipeline is executed through an accessible terminal command-line interface (CLI), where a single YAML configuration file defines molecular composition, box geometry, CG model, AA force field, salt concentration, and other simulation parameters. Figure S1 shows the corresponding command sequence, representative YAML fields, and output organization.

**Figure 1:**
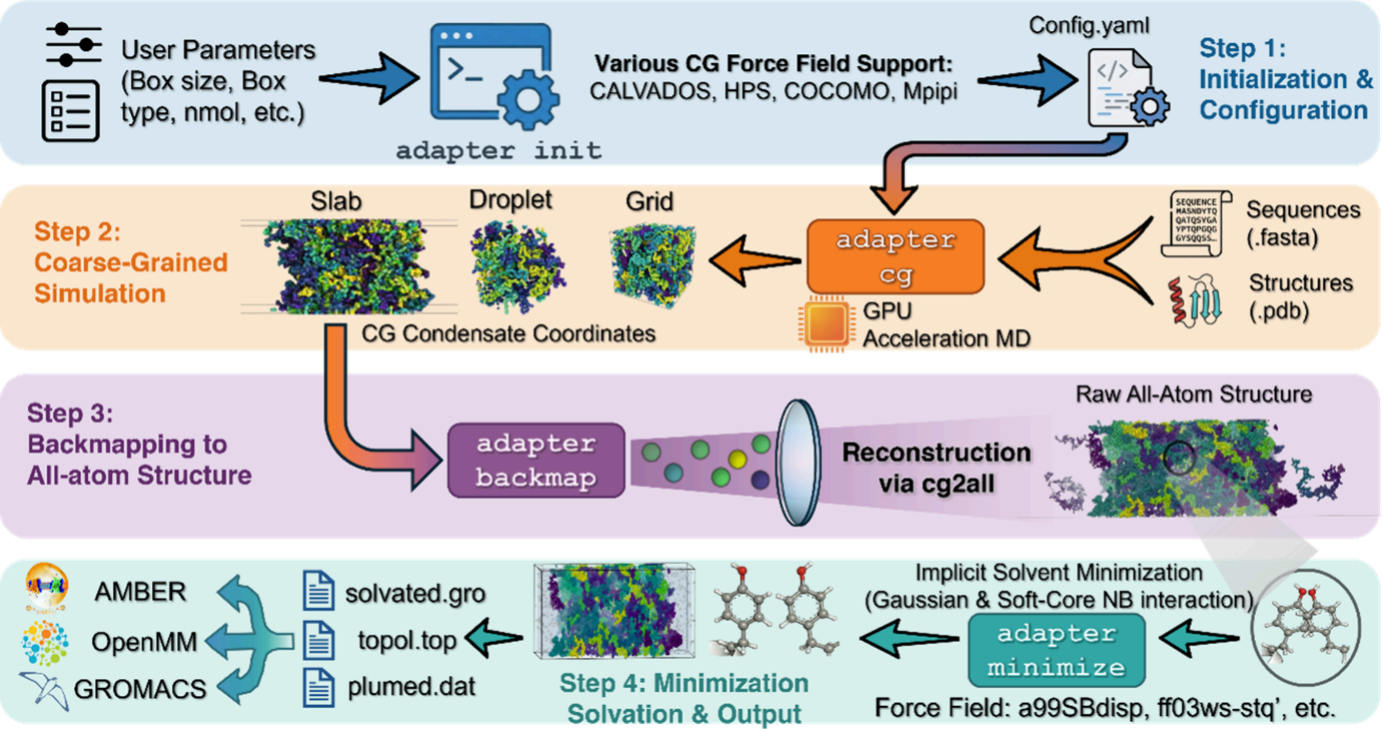
Overall CG-to-AA simulation workflow of **CondenSimAdapter**.

Internally, the CG stage provides OpenMM-native implementations of CALVADOS,^33,34^ HPS-Urry,^35^ COCOMO2,^36^ and Mpipi-Recharged^37^ under a unified data model, offering preconfigured system geometries (slab, droplet, or cubic) with an optional entanglement check.^38^ Consistency with reference CG implementations is summarized in Figure S2. The backmapping stage embeds the cg2all SE(3)-Transformer model to reconstruct AA coordinates from one-bead CG input, followed by PDB standardization for topology generation. Because cg2all reconstructs coordinates but does not by itself relax unfavorable dense-phase interchain contacts, **CondenSimAdapter** applies Gaussian repulsive potential,^39^ soft-core potential,^40^ and standard nonbonded potential minimization in OBC2 ^41^ Generalized Born implicit solvent, followed by explicit solvation and ionization. For multidomain proteins (MDPs), **CondenSimAdapter** also generates native-contact *Q* restraints^42^ to protect folded domains during early equilibration, implemented through PLUMED.^43^ Additional methodological details on CG implementation, coordinate reconstruction, staged relaxation, folded-domain restraints, simulation protocols, and entanglement detection are provided in SI Sections S2–S6 and S11.

We evaluated **CondenSimAdapter** across an extensive validation set, spanning from fully disordered homotypic condensates to multicomponent mixtures and complex multidomain assemblies, tested under multiple CG and AA force-field combinations (Table 1). Detailed sequences, definitions of domains, and full names of abbreviated proteins are provided in the Supporting Information (Tables S1, S2, and S6).

**Table 1:** Summary of Simulation Systems

**system**	**Nmol**	**CG force field**	**all-atom force field**
FUS LC	60	CALVADOS	ff99sb-STQp
		COCOMO2	
		Mpipi-Recharged	
		HPS-Urry	

H1-ProTalpha	24, 20	Mpipi-Recharged	a99SBdisp
			ff03w-sc
			ff99sbws-STQp
			ff99sbws-STQ
			des-amber
			des-amber-sf1.0
			ff99sb-ildn
			ff14sb
			CHARMM36m

LAF1 RGG	60	CALVADOS	ff99sbws-STQp
TDP43 LCD	68		
DDX4 LCD	43		
(GRGDSPYS)_25_	50		
A1_LCD	75		
hnRNPA1S	32		
TDP43	25		
FUS	20		
			
FUS LC	40	Mpipi-Recharged	ff99sbws-STQp

## CondenSimAdapter Robustly Resolves Structural Conflicts of Backmapped Structures

A key prerequisite for a reliable multiscale workflow is the systematic elimination of structure conflicts introduced during the resolution transformation. We evaluated whether **CondenSimAdapter** produces production-ready AA condensate configurations by examining: (1) whether major structure conflicts, including severe steric clashes, ring penetrations, and chirality errors (see SI Section S7 for detailed definitions and metrics), are removed;^22^ whether the system energy is minimized to reach a practical convergence threshold (*F*_max_ < 500 kJmol^–1^nm^–1^); (2) whether the resulting systems remain stable during short AA production tests. For each system, five independent repeats were generated from distinct equilibrated CG snapshots and processed through the full backmapping and minimization workflow. To quantify the structural conflicts, we defined three metrics: (1) steric clashes (non-hydrogen atoms closer than 1.2 Å, excluding bonded neighbors), (2) ring penetrations (covalent bonds passing through aromatic or cyclic rings), and (3) chirality errors (incorrect handedness at chiral centers).

We first examined the conflict-resolving ability of **CondenSimAdapter** by constructing condensate models for a wide spectrum of proteins ranging from diverse intrinsically disordered proteins (IDPs) to densely packed MDPs. After **CondenSimAdapter** minimization, all clash rates (initially 1.1–7.1% across systems) and nearly all ring-penetration rates (initially 0.2–3.1%) were reduced to zero, except for the TDP43 condensate, which retained a residual ring-penetration rate of 0.01%. Furthermore, no chirality errors were detected across the entire benchmark set, either in the initial backmapped snapshots or after final minimization. Final maximum forces for all tested systems were below the practical minimization threshold (Table S3), interchain pair-distance redistribution during replicate minimization is shown in Figure S4, and five-repeat 10 ns NPT tests showed stable relative potential energy evolution (Figure S6). In contrast, direct standard minimization of crowded backmapped structures can remain above 10̂5 kJ mol^–1^ nm^–1^, highlighting the role of the staged optimization protocol.

We also tested the ability of **CondenSimAdapter** to rectify structural conflicts in structures reconstructed by the four CG models. As shown in Figure 2b, for our model condensate system formed by FUS LC, the initial configurations backmapped from structures generated by these CG models exhibited ∼2% steric clashes and ∼0.5% ring penetrations. While these percentages may appear small, in a large condensate system comprising nearly ten thousand residues, they translate into hundreds of steric clashes and dozens of ring penetrations, more than enough to cause conventional energy minimization to fail. Following the **CondenSimAdapter** minimization protocol, these conflicts were uniformly eliminated, yielding physically plausible geometries ready for MD integration.

**Figure 2:**
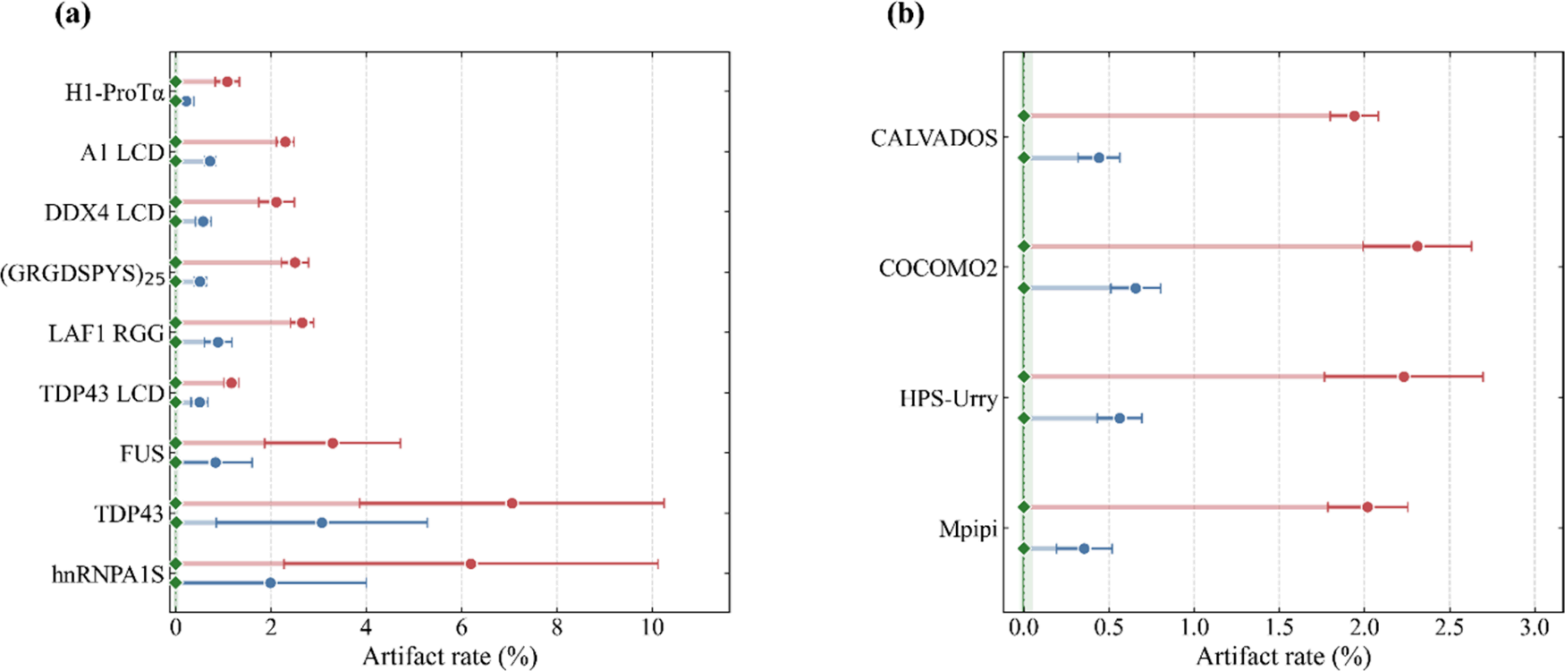
Removal of backmapping artifacts by energy minimization across protein condensate systems. (a) H1-ProTalpha, IDP condensates, and MDP condensates. (b) FUS LC reconstructed from four CG force fields. Red circles denote backmapped steric-clash rates, blue circles denote backmapped ring-penetration rates, and green diamonds denote postminimization residuals. Values are mean ± SD over five independent repeats. Numerical source data are provided in Table S4.

## Structural Integrity of Folded Domains in Condensates

For condensates containing folded domains, it is also crucial that the structures of the folded domains are conserved in the highly crowded condensate models generated by multiscale approaches. To achieve this goal, **CondenSimAdapter** uses native contacts (*Q*) of folded domains as order parameters and applies *Q*-based restraints, as detailed in SI Sections S6 and S8, to maintain folded domains during preparatory equilibration.^42^ We evaluated the performance of this approach for the H1-ProTalpha condensate system, which serves as a representative heterotypic MDP/IDP condensate where disordered protein ProTalpha coexists with H1 protein containing a folded winged-helix globular domain.^16^

Figure 3a shows a representative all-atom H1-ProTalpha condensate generated by **CondenSimAdapter** after preparatory equilibration. To evaluate folded-domain stability across AA model choices, we analyzed the H1 backbone RMSD across nine force fields during subsequent production simulations without any restraints (Figure 3b). The mean RMSD values remained low (<0.16 Å) and tightly distributed across both AMBER-family models and CHARMM36m, indicating that the structural integrity of the folded H1 domain is well-preserved. A control simulation without preparatory *Q*-restrained equilibration showed an increase in domain RMSD in production runs (Figure S5), confirming the necessity of our protective restraints during initial relaxation.

**Figure 3:**
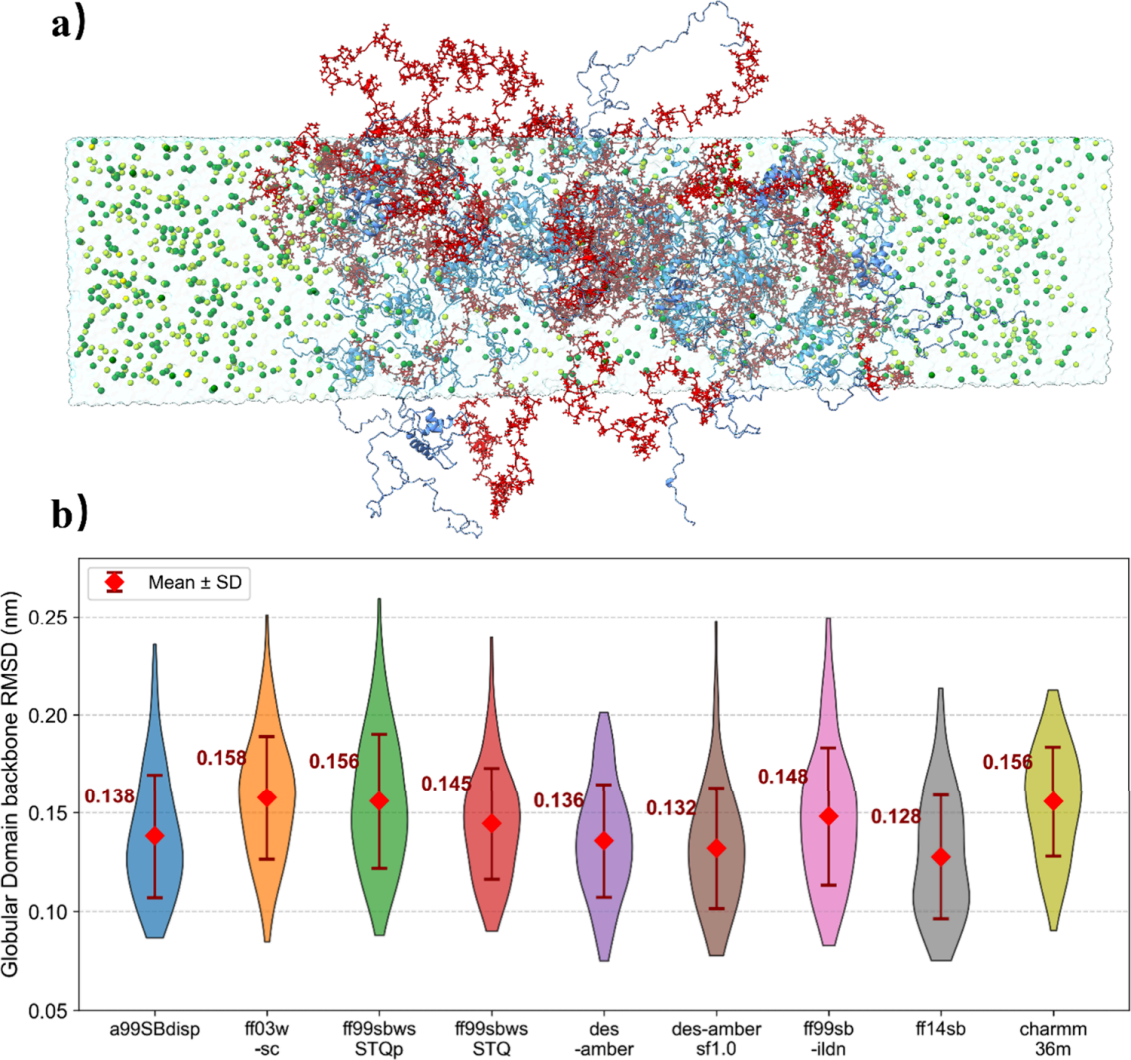
H1 Domain structural stability across all-atom force fields. (a) Representative all-atom H1-ProTalpha condensate. H1 are shown in blue, ProTalpha in red, water as a semitransparent cyan surface, Na^+^ as yellow spheres, and Cl^–^ as green spheres. (b) H1 globular-domain backbone RMSD distributions across nine AA force fields, calculated over the final 5 ns of 10 ns production trajectories. Each violin contains 100 per-domain mean RMSD values per force field from five independent repeats and 20 H1 domains per repeat. Red diamonds and error bars indicate mean ± SD.

## CondenSimAdapter-Generated Coordinates Allow for Reproduction of Physical Observables of Condensates

We further assessed whether the configurations produced by **CondenSimAdapter** correspond to physically realistic ensembles suitable for reproducing physical properties of condensates. To this end, we conducted an extensive 2 μs production simulation of a 40-chain FUS LC condensate (detailed simulation setup in SI Section S10), a model system that has been well-characterized by abundant experimental data^44,45^ and previously reported atomistic simulations,^14^ enabling direct comparisons with our results.

The generated structural ensemble agreed well with established FUS LC reference behavior (Figure 4). Specifically, the simulation maintained a stable condensed-phase protein density of 528 mg/mL and a water density of 615 mg/mL. These bulk values are consistent with previously reported atomistic simulations (approximately 477 and 600 mg/mL, respectively),^14^ indicating that the reconstructed condensate maintained a realistic protein packing and hydration throughout the 2 μs atomistic simulation. The ion density profile further showed clear phase partitioning, with higher ion density in the dilute phase and lower ion density within the dense phase, consistent with previously reported ion partitioning behavior in FUS LC condensates.^14^

**Figure 4:**
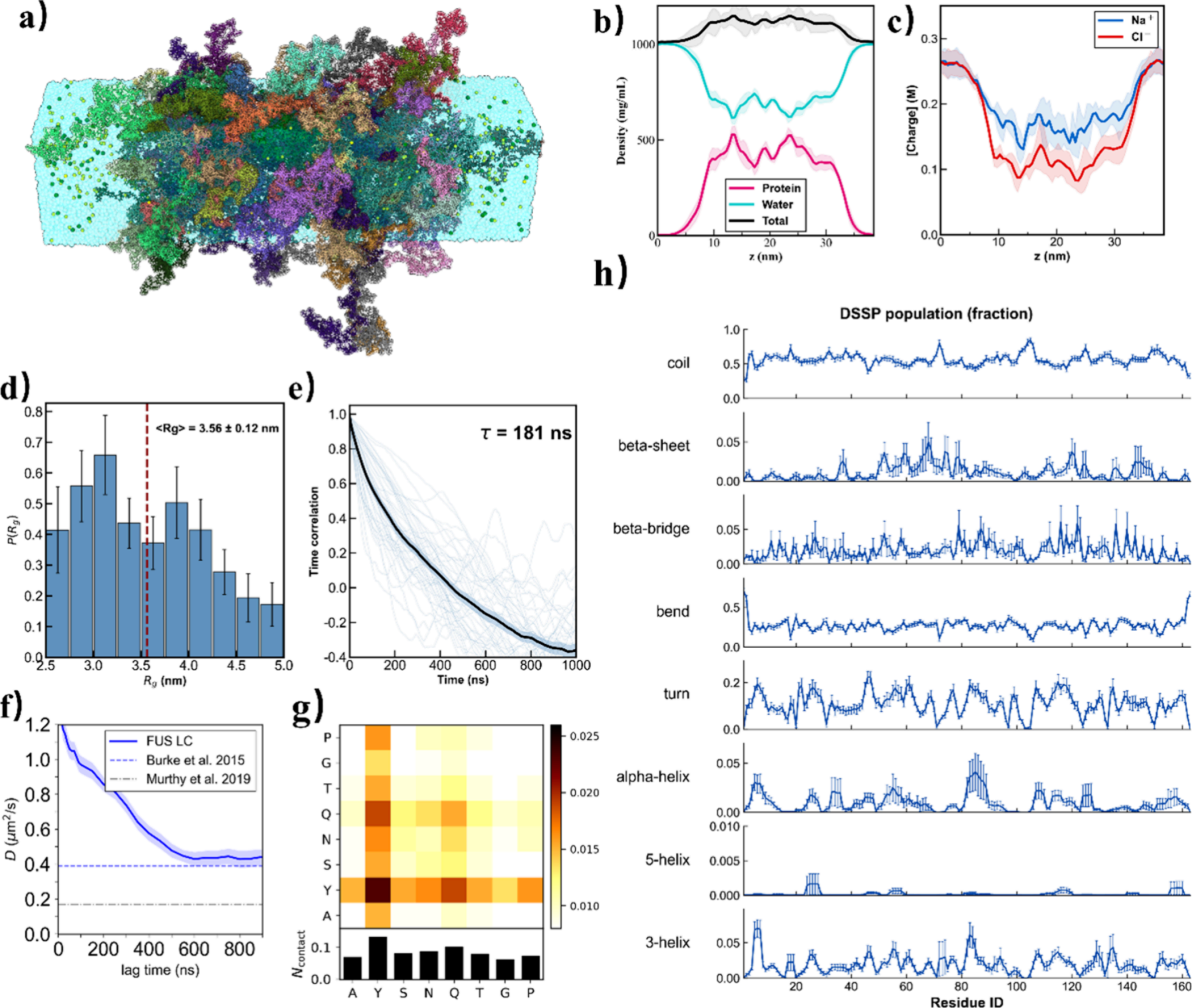
Structural, dynamical, and interaction properties of the 40-chain FUS LC condensate. The system was generated from Mpipi-Recharged CG sampling, cg2all backmapping, **CondenSimAdapter** minimization, ff99sbws-STQp protein parameters,^29^ and TIP4*P*/2005 water,^46^ followed by a 2 μs AA validation simulation. (a) Representative all-atom snapshot of the solvated slab. (b) Protein, water, and total mass density profiles along the slab normal (*z*). (c) Na^+^ and Cl^–^ density profiles along *z*. (d) Chain *R*_g_ probability distribution. (e) *R*_g_ time autocorrelation function. (f) Slab-normal diffusion coefficient from propagator analysis. (g) Intermolecular amino-acid-type contact matrix. (h) Per-residue DSSP secondary-structure populations. In panels d, e, and h, error bars or shaded bands denote SEM across 40 chains where applicable. In panels b and c, shaded bands denote standard deviations across trajectory blocks. In panel f, the shaded band denotes chain-bootstrap SD from resampling the 40 chain COM trajectories.

We next analyzed single-chain dimensions and mobility. The mean chain radius of gyration (*R*_g_) was 3.56 nm, and its conformational relaxation time (τ) was 181 ns, consistent with previously reported atomistic simulations.^14^ Furthermore, propagator analysis along the slab normal yielded a diffusion coefficient of 0.45 μm^2^ s^–1^, consistent with reference dynamics.^45^ This confirms that the reconstruction process introduces no artificial kinetic trapping, allowing chains to diffuse realistically.

Finally, we investigated the intramolecular conformational preferences and intermolecular contact networks that characterize the FUS LC condensed phase. Secondary structure analysis (DSSP) confirmed that the chains remained predominantly disordered, with low α-helix and β-sheet fractions. In addition, the pattern of intermolecular contacts was enriched in Tyr- and Gln-mediated interactions, faithfully reproducing the molecular grammar of FUS LC assembly.^44,45^ Crucially, the close agreement between these calculated multiscale observables and reference benchmarks confirms that **CondenSimAdapter** successfully bridges the resolution gap, generating physically realistic all-atom ensembles that retain the thermodynamic and dynamic integrity of the condensed phase over long-timescale simulations.

## Conclusions and Outlook

In this work, we developed **CondenSimAdapter** to address the practical barriers posed by the “relaxation bottleneck” and force-field diversity in multiscale condensate simulations. This tool combines a physics-inspired optimization protocol with a unified model interface, greatly simplifying what was once an error-prone manual procedure into a robust, high-throughput pipeline. Validation across diverse condensate systems confirmed that the workflow reliably removes major structure conflicts and yields physically realistic ensembles.

**CondenSimAdapter** is therefore complementary to existing related tools such as CHARMM-GUI Multicomponent Assembler,^47^ IPAMD,^48^ and OpenABC;^49^ a detailed comparison is provided in Table S5 and SI Section 12. **CondenSimAdapter** fills the CG-sampling-to-AA-production step: it generates a pre-equilibrated dense protein condensate, rebuilds it at atomistic resolution, and overcomes the dense-phase relaxation bottleneck while allowing CG and AA force-field choices to be varied in a controlled workflow.

The present implementation also defines clear directions for extension. Beyond proteins, nucleic acids are among the most important biomolecular components of condensates.^50^ The current workflow is protein-centered and uses cg2all as the default reconstruction engine, so nucleic-acid-containing condensates will require additional CG representations, topology handling, and backmapping strategies.^51^ CG-generated dense phases can also contain chain entanglements, especially in systems that form solid-like or gel-like condensates experimentally, and such topological problems are difficult to resolve after conversion to an AA representation. **CondenSimAdapter** therefore includes an entanglement-detection step within the workflow, allowing elevated-entanglement CG configurations to be flagged before AA production setup; the detector comparison with Z1+^52^ (an established algorithm for primitive path analysis and entanglement quantification) is shown in Figure S7. Support for machine-learning force fields such as SO3LR^53^ and MACE-OFF^54^ is another direction for improving interaction accuracy.

By standardizing input and output interfaces and automating the resolution transition, **CondenSimAdapter** reduces the technical overhead of multiscale modeling, enabling routine atomistic validation of coarse-grained models. We expect **CondenSimAdapter** to become a useful tool for multiscale investigations of biomolecular phase separation.

## Supplementary Material



## Data Availability

The software used in this study,**CondenSimAdapter**, along with its installation guide and tutorials can be accessed at https://github.com/hanlab-computChem/CondenSimAdapter. All essential simulation data for the systems discussed in Table 1 are available at https://zenodo.org/records/20019176. Analysis scripts, plotting scripts, and simulation trajectories can be obtained upon request from the corresponding authors.

## Notes

Workflow illustrations and details; CG model implementation details; CG and AA MD simulation protocols; details of native-contact Q-restraint and entanglement-detection algorithms; analysis methods; related-workflow comparisons; supplementary figures and tables; and amino-acid sequences of the systems studied (PDF)
