# Supplementary material for: CondenSimAdapter: A Versatile Builder for Multiscale Simulations of Protein Condensates with Broad Force-Field Compatibility and Robust Dense-Phase Relaxation
Source: J Chem Inf Model. 2026 Jun 30;66(13):7337–44. doi: 10.1021/acs.jcim.6c00413 (PMC13370849; doi:10.1021/acs.jcim.6c00413)
Supplement: Supplementary file 1 [file ci6c00413_si_001.pdf]

CondenSimAdapter: A versatile builder for multiscale simulations of protein condensates with broad force-field compatibility and robust dense-phase relaxation

Xiaojing Tian[a,d], Wei Han[b,c,d,*]

[a] School of Chemical Biology and Biotechnology, Peking University Shenzhen Graduate School, Shenzhen 518055, China

[b] Department of Chemistry, Faculty of Science, Hong Kong Baptist University, Hong Kong SAR, China

[c] Institute for Research and Continuing Education (Shenzhen), Hong Kong Baptist University, Shenzhen 518000, China

[d] Institute of Chemical Biology, Shenzhen Bay Laboratory, Shenzhen 518132, China

[*]Corresponding author, E-mail: hanw_chem@hkbu.edu.hk (W. H.)

Supporting Information

Supporting Information Contents

S1. Software workflow and generated files S3

S2. CG model implementations and consistency checks S5

S3. Coordinate generation and cg2all backmapping details S6

S4. Three-stage minimization protocol S8

S5. Detailed MD simulation protocols S11

S6. Native-contact restraints for folded domains S14

S7. Structural quality metrics S16

S8. Statistical analysis and independent repeats S17

S9. Energy stability after construction S19

S10. FUS LC 2 μs atomistic validation setup and analysis details S20

S11. Entanglement detection and Z1+ benchmark S22

S12. Comparison with related workflows S25

S13. Supplementary tables S27

Reference S30

S1. Software workflow and generated files

CondenSimAdapter is operated through a few streamlined commands corresponding to the main workflow stages. The workflow begins with adapter init, which generates a template YAML configuration file specifying key parameters such as protein components, copy numbers, box geometry, and force fields To ensure consistency, this single configuration file is shared across subsequent adapter cg, adapter backmap, and adapter minimize commands.

The adapter cg stage generates the CG topology, places molecules in the selected geometry, and runs CG sampling using the OpenMM-native implementation. We primarily support slab, droplet, and cubic/grid placements; for the condensates studied here, we used slab geometries. Outputs from this stage include the final CG coordinates (final.pdb or equivalent), CG trajectories, sequence/chain metadata, and force-field-specific intermediate files needed for reproducibility.

The adapter backmap stage converts a selected CG snapshot into all-atom (AA) coordinates using the embedded cg2all model. The backmapped PDB is then standardized for downstream topology generation through corrections to chain identifiers, residue numbering, atom names, terminal records, and model/frame handling. Because this reconstruction step is modular, while we used cg2all here, other CG-to-AA methods could be seamlessly integrated in the future.

The adapter minimize stage generates force-field-specific topology files, applies the **CondenSimAdapter** staged relaxation protocol, and constructs solvated/ionized AA systems.

The principal outputs include the minimized dry structure, solvated structure (`solvated.gro`), GROMACS topology (`topol.top`), ionized production input files, and optional `plumed.dat` files for folded-domain restraints. When requested, output systems can be exported for use with GROMACS,[1] OpenMM,[2] or Amber[3] workflows.

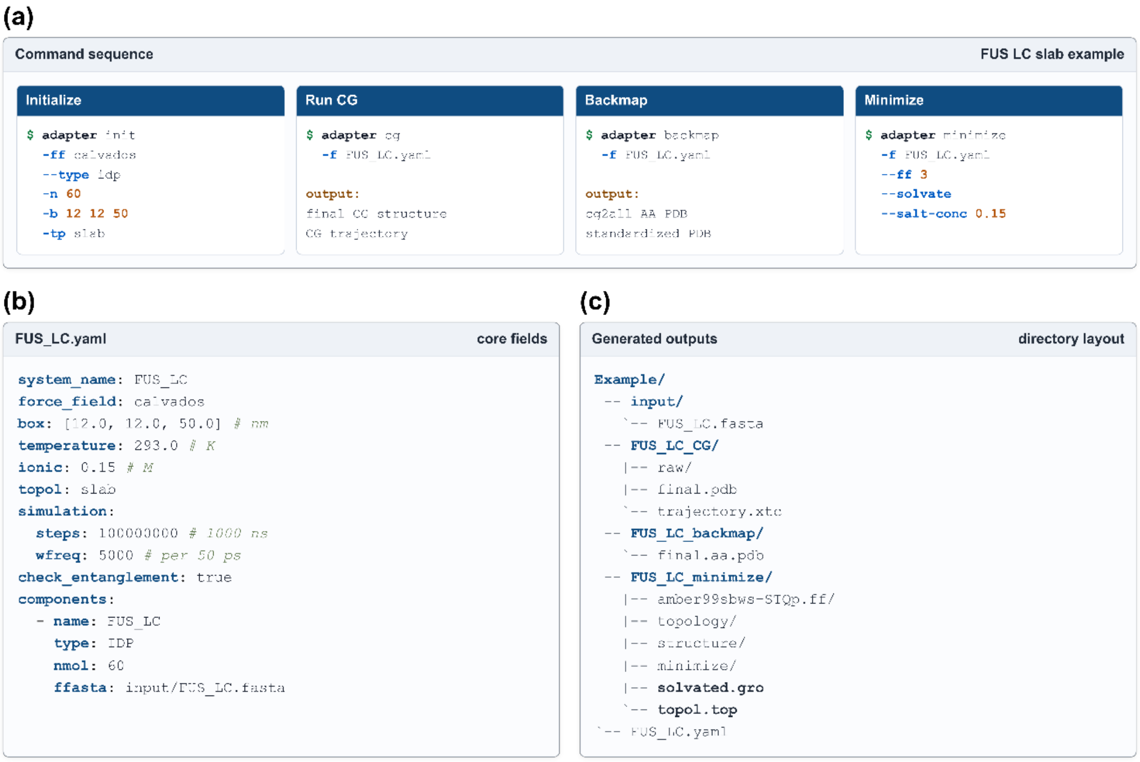

Figure S1. Detailed execution and output organization of CondenSimAdapter. The example illustrates construction of a FUS LC slab system from sequence information. (a) The command sequence first creates a YAML configuration file with `adapter init` and then reuses the same configuration for `adapter cg`, `adapter backmap`, and `adapter minimize`. These steps perform CG sampling, cg2all-based reconstruction, staged relaxation, solvation, ionization, and topology generation. (b) Representative YAML fields define the condensate geometry, molecular components, copy numbers, CG model, salt concentration, and simulation settings. (c) The resulting directory organization separates CG trajectories, backmapped coordinates, minimized dry structures, solvated/ionized AA systems, topology files, and optional restraint files for downstream MD simulations.

S2. CG model implementations and consistency checks

CondenSimAdapter provides new OpenMM-native implementations for four residue-resolution CG force fields: CALVADOS4,5, COCOMO26, Mpipi-Recharged7, and HPS-Urry8. By unifying these models under a single data structure, the workflow seamlessly manages system composition, trajectories, restart files, and backmapping inputs regardless of the chosen force field. We retained the original functional forms and parameters wherever possible, while exposing force-field-specific options through the shared YAML configuration.

Because each CG model treats multidomain proteins differently, CondenSimAdapter is designed to preserve these specific characteristics. In COCOMO2 and Mpipi-Recharged, interactions involving structured domains are scaled according to the original model rules. In CALVADOS3, folded domains are represented by center-of-mass beads and restrained with elastic-network interactions. HPS-Urry originally treats folded domains as rigid bodies through HOOMD-blue9 rigid-body constraints. Since OpenMM lacks an exact equivalent for rigid-body constraints in this context, CondenSimAdapter represents HPS-Urry folded domains using an elastic network with the same cutoff and force-constant convention used for CALVADOS folded-domain handling (0.9 nm cutoff and 700 kJ mol^{-1} nm^{-2} force constant). This minor adjustment accounts for the small HPS-Urry folded-domain difference noted below

As a consistency check for the unified engine, we compared R_g distributions generated by **CondenSimAdapter** against the corresponding published or reference implementations.

Jensen-Shannon (JS) divergence values were small for most systems, typically 0.0028-0.0051. The largest deviation was observed for the H1 system under HPS-Urry (JS = 0.0174), consistent with the different folded-domain treatment described above.

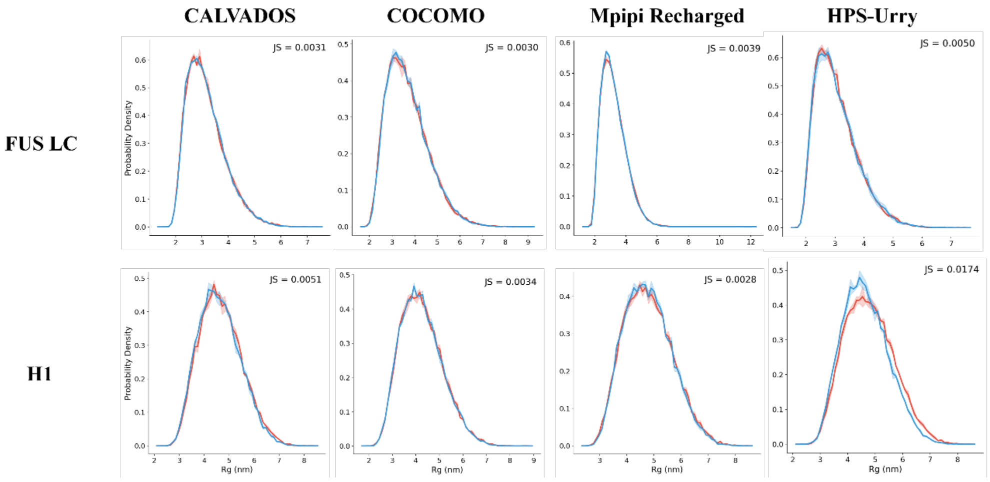

Figure S2. CG model consistency check by R_g distributions. Radius-of-gyration distributions generated by the **CondenSimAdapter** OpenMM-native implementations are compared with distributions from the corresponding reference implementations. Red curves denote **CondenSimAdapter** results, and blue curves denote the references: CALVADOS, COCOMO2, OpenMpipi/Mpipi-Recharged, and HOOMD-blue plus azplugins for HPS-Urry. The Jensen-Shannon (JS) divergence is reported in each panel to quantify distributional agreement. Most comparisons give small JS values (0.0028-0.0051), indicating that the unified implementation reproduces the reference conformational ensembles. The largest difference occurs for H1 under HPS-Urry (JS = 0.0174), consistent with the elastic-network folded-domain treatment used here instead of the rigid-body treatment in the HOOMD-blue reference.

S3. Coordinate generation and cg2all backmapping details

Initial CG coordinates are generated from sequence and optional domain-structure inputs. For IDP-only systems, each protein chain is initialized from the sequence and placed according to the requested geometry. For MDP systems, folded-domain coordinates are read from supplied PDB files or predicted structures, while disordered linkers are generated from

sequence. After propagating the CG trajectory for the prescribed number of steps, selected snapshots are extracted for backmapping.

CondenSimAdapter uses cg2all[10] as the default CG-to-AA reconstruction engine. cg2all is an $SE(3)$-Transformer-based model that reconstructs all-atom protein coordinates from one-bead-per-residue representations. In this work, the pretrained Cα and center-of-mass one-bead models were used to reconstruct individual condensate snapshots selected from the CG trajectories.

The cg2all output is post-processed before topology generation. This includes standardizing residue and atom names, assigning chain identifiers, correcting residue numbering across multichain condensates, and removing records that are incompatible with force-field-specific topology generation. Protonation states and terminal states are then assigned during AA topology construction rather than inside cg2all.

Using cg2all introduces certain constraints to our workflow. Since it was trained mostly on PDB structures, the model carries a structural bias toward folded proteins. Additionally, the default model generates a deterministic reconstruction and cannot sample rotamer or protonation-state ensembles. While cg2all accurately rebuilds local geometries in dense condensates, it cannot automatically resolve steric clashes caused by crowded CG packing or periodic boundaries. This highlights why a dedicated downstream relaxation step is crucial.

S4. Three-stage minimization protocol

Backmapped condensate structures often exhibit severe atomic overlaps that cause standard energy minimization to fail. To resolve this, CondenSimAdapter employs a staged relaxation protocol before setting up the explicit-solvent production run. The protocol uses the OBC211 Generalized Born implicit-solvent model. Bonded terms are kept unchanged, whereas nonbonded interactions, including 1-4 interactions, are modified during the early relaxation stages.

In the first stage, we introduce a short-range Gaussian repulsion to gradually eliminate extreme atomic overlaps.[12]

$$E_{\mathrm{gauss}}(r) = g_h \exp[-(r/g_w)^2].$$

The default parameters used in this work were $g_w = 1.1$ nm and $g_h = 8000$ kJ mol^{-1}. This term primarily acts on atom pairs at extremely short distances and avoids the singular gradients that would occur if the full Lennard-Jones potential were applied immediately.

After the most severe overlaps are removed, **CondenSimAdapter** switches to the linear-force soft-core potential.[13] In this scheme, the standard force is used beyond a switching distance, whereas the short-range force is replaced by a finite linear expansion:

$$F_{\mathrm{soft}}(r) = \begin{cases} F(r), & r > r_{\mathrm{sw}}, \\ F(r_{\mathrm{sw}}) + F'(r_{\mathrm{sw}})(r_{\mathrm{sw}} - r), & r \le r_{\mathrm{sw}}. \end{cases}$$

Separate switching distances are defined for Lennard-Jones and Coulomb interactions:

$$r_{\mathrm{sw}}^{\mathrm{LJ}} = 2^{1/6}\sigma\alpha_{\mathrm{LJ}}(1-\lambda)^{1/6},$$

$$r_{\mathrm{sw}}^{\mathrm{Coul}} = \alpha_{\mathrm{Coul}}(1 + |q_1 q_2|)(1-\lambda)^{1/6}.$$

Here, σ is the Lennard-Jones size parameter, q_1 and q_2 are partial charges, and the softness parameters were $\alpha_{\mathrm{LJ}} = 0.85$ and $\alpha_{\mathrm{Coul}} = 0.30$. The shared exponent of 1/6 ensures that Lennard-Jones and Coulomb switching points soften and recover at the same rate as λ is varied, following the original Gapsys formulation. In this work, λ is increased from softer to harder states through the schedule $0.75 \rightarrow 0.85 \rightarrow 0.95$, progressively restoring the standard nonbonded interaction.

The final stage uses the standard force-field nonbonded potential to relax the structure close to the physical Hamiltonian. Each soft-core stage used a 2.0 nm nonbonded cutoff, a force-convergence threshold of 100 kJ mol-1 nm-1, and at most 5000 optimization iterations. After the staged dry-structure relaxation, systems were solvated and then subjected to steepest-descent and conjugate-gradient minimizations as described in SI Section S5.

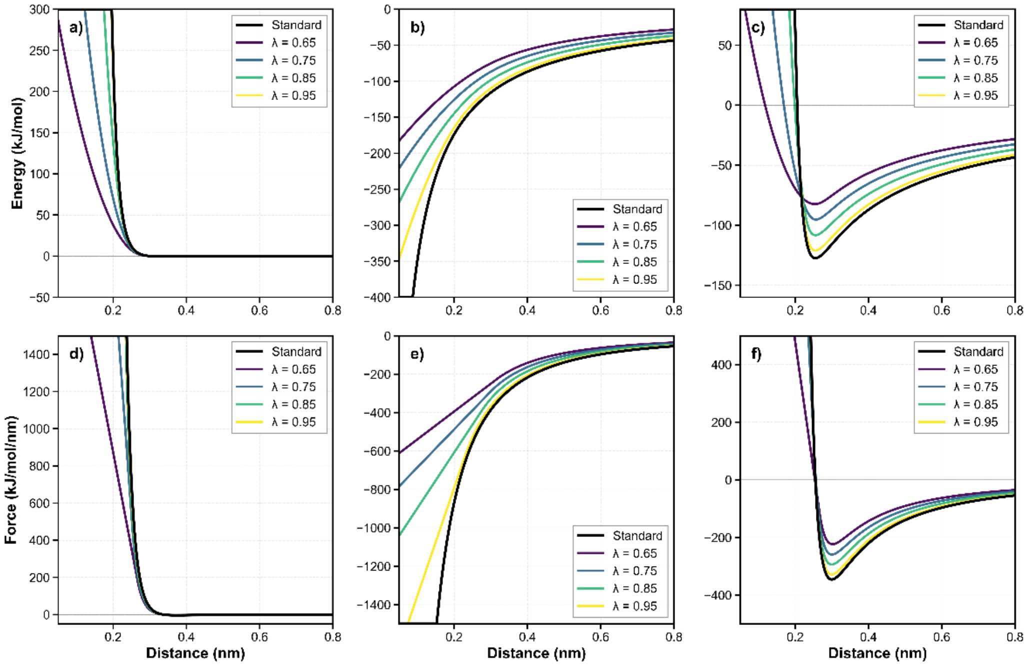

Figure S3. GAPSYS soft-core treatment removes short-range singularities in nonbonded potentials and forces. Standard and soft-core nonbonded interactions are shown for a representative atom pair with $\sigma = 0.30$ nm, $\epsilon = 0.50$ kJ mol^{-1}, $q_1 = +0.5e$, and $q_2 = -0.5e$. Panels (a-c) show the Lennard-Jones, Coulomb, and total nonbonded potentials, respectively; panels (d-f) show the corresponding forces. Black curves denote the standard nonbonded interaction, and colored curves denote soft-core interactions at different λ values. The soft-core formulation keeps both potentials and forces finite at short distances.

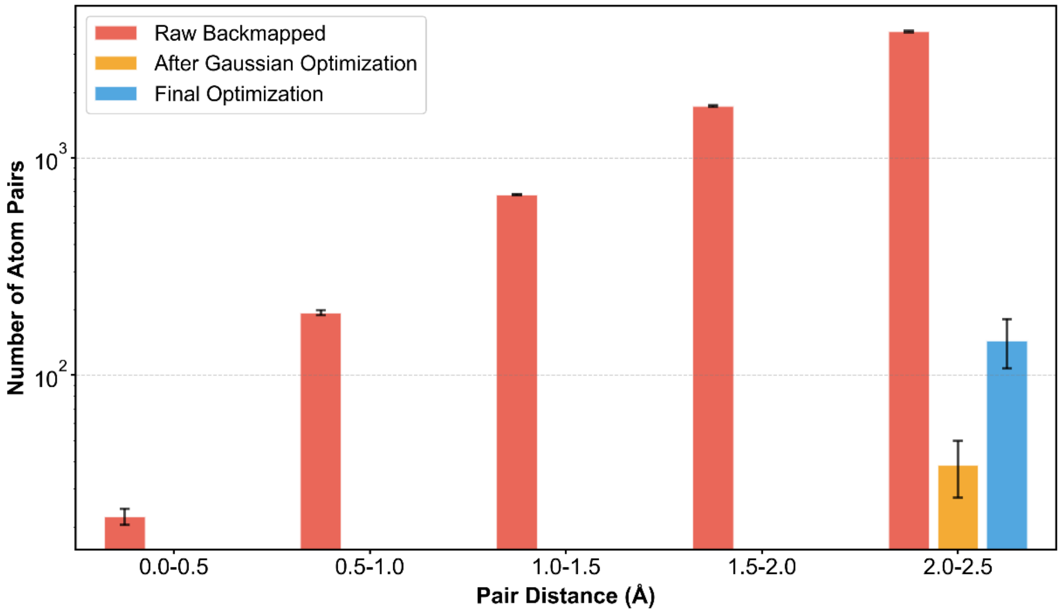

Figure S4. Interchain pair-distance redistribution during replicate minimization. FUS LC replicate backmapping and minimization cycles are compared across raw cg2all output,

Gaussian/soft-core relaxation, and final standard-potential minimization. Distances below 3.5 Å are binned into 0.5 Å intervals and reported as mean ± SD across five repeats. The disappearance of the shortest-distance bins reflects removal of severe overlaps, whereas the remaining 1.5-3.5 Å population after standard minimization reflects recovery of physically plausible close contacts rather than unresolved severe clashes.

S5. Detailed MD simulation protocols

S5.1 CG simulation protocol

All CG simulations were performed using **CondenSimAdapter** with the OpenMM-native implementation. CALVADOS versions were selected according to system composition: CALVADOS2-like settings for IDP-only systems and CALVADOS3-like folded-domain handling for MDP systems. HPS-Urry, COCOMO2, and Mpipi-Recharged used the model-specific interaction forms described in SI Section S2.

Unless otherwise stated, CG simulations were performed in the NVT ensemble at 300 K with 0.15 M monovalent salt. Temperature was maintained with Langevin dynamics using a friction coefficient of 0.01 ps^{-1} and a 10 fs timestep. The CG production length was 1 μs. Force-field-specific nonbonded cutoffs followed the original model recommendations and were generally near 2.0 nm for pair interactions.

S5.2 Backmapping and dry-structure minimization

While users can supply specific snapshots, CondenSimAdapter defaults to using the final frame of the CG production trajectory for backmapping. The selected CG structure is then reconstructed with cg2all, and the resulting AA coordinates serve as the basis for generating force-field-specific topologies. Dry structures are relaxed in OBC2 implicit

solvent using the CondenSimAdapter Gaussian/soft-core/standard protocol described in SI Section S4.S5.3 Solvation, ionization, and solvated-system minimization

After dry-structure relaxation, explicit solvent and ions were added to produce systems containing 0.15 M NaCl plus neutralizing counterions. Unless otherwise stated, the 10 ns construction tests used the water-model choices described in SI Section S5.4. The 2 μs FUS LC validation used TIP4P/2005 water[14] as described in SI Section S10.

Solvated systems were further minimized in two stages. A steepest-descent minimization was first applied for up to 5000 steps with a force-convergence threshold of 2000 kJ mol^{-1} nm^{-1}. This was followed by conjugate-gradient minimization for up to 5000 steps with a stricter threshold of 500 kJ mol^{-1} nm^{-1}. Electrostatics were treated with particle-mesh Ewald (PME),[15] and both Coulomb and van der Waals interactions used 1.0 nm real-space cutoffs. No bond constraints were applied during minimization.

S5.4 Short AA production tests

Short AA production tests were performed with GROMACS 2025. The H1-ProTalpha force-field comparison used nine registered AA force fields, in the order ff1-ff9: a99SBdisp,[16] ff03w-sc,[17] ff99sbws-STQp,[17] ff99sbws-STQ,[18] des-amber,[19] des-amber-sf1.0,[19] ff99sb-ildn,[20] ff14sb,[21] and CHARMM36m.[22] The first six force fields used their recommended TIP4P or TIP4P-variant water models, whereas the last three used TIP3P for Amber and TIP3P-modified for CHARMM 36m.

Equilibration and production were performed in the NPT ensemble at 300 K and 1 bar. Temperature was controlled with the velocity-rescale thermostat[23], and pressure was controlled using the stochastic cell-rescaling[24] barostat with a coupling time of 0.5 ps and an isothermal compressibility of 4.5×10^{-5} bar^{-1}. Electrostatics were treated with PME, Coulomb and van der Waals interactions used 1.0 nm real-space cutoffs, long-range dispersion corrections were applied to energy and pressure, and hydrogen-bond constraints were enforced with LINCS. The initial NPT equilibration stage lasted 100 ps with a 0.5 fs timestep. IDP-only systems then proceeded directly to 10 ns NPT production with a 2 fs timestep. Folded-domain-containing systems underwent the same 100 ps equilibration, followed by 1 ns of Q-restrained NPT equilibration and 10 ns of unrestrained NPT production. The Q-restraint stage is described in SI Section S6.

For IDP-only, MDP system-diversity, and H1-ProTalpha tests, production runs used the C-rescale barostat. CHARMM36m was run with the same 1.0 nm cutoffs as the Amber-family force fields for the short production tests; this choice was made for comparison consistency and does not affect the structural artifact metrics reported here.

S5.5 Independent repeats

For FUS LC CG-force-field tests, each of the five repeats for each CG model was initiated from a distinct CG output configuration; unless a snapshot was explicitly specified, **CondenSimAdapter** selected the final CG frame for backmapping. H1-ProTalpha, IDP condensates, and MDP condensates followed the same repeat logic. IDP-only repeats were constructed by CG configuration generation, backmapping, three stage minimization, explicit

solvation and ionization, solvated-system minimization, 100 ps NPT equilibration, and 10 ns NPT production. Folded-domain-containing repeats used the same construction sequence, followed by 100 ps NPT equilibration, 1 ns Q-restrained NPT equilibration, and 10 ns unrestrained NPT production.

S6. Native-contact restraints for folded domains

For MDP condensates, **CondenSimAdapter** generates Q-based restraints to preserve folded-domain integrity during early AA equilibration.[25,26] A PDB structure, or a predicted structure when no experimental structure is available, is provided for each folded domain. **CondenSimAdapter** identifies all heavy-atom native contacts within folded domains using a 0.45 nm cutoff and a sequence-separation filter of $|i - j| > 3$ residues. Contacts involving hydrogen atoms are excluded.

For a folded domain with N native contacts, the native-contact fraction is:

$$Q = \frac{1}{N} \sum_{(i,j) \in S} \frac{1}{1 + \exp[\beta(r_{ij} - r_{ij}^0)]}.$$

Here, S is the native-contact set, r_{ij}^0 is the native distance in the reference structure, and β controls the switching sharpness. The resulting Q collective variable was applied as a PLUMED-compatible restraint for each folded domain. During folded-domain equilibration, each domain Q value was restrained by:

$$V(Q_i) = \frac{1}{2} k (Q_i - 1)^2.$$

The force constant used in the H1-ProTalpha and MDP tests was $k = 1000$ kJ mol^{-1}. The restrained stage lasted 1 ns for folded-domain-containing systems, followed by the unrestrained 10 ns production tests described in SI Section S5. IDP-only systems did not use this stage.

To test the protective role of the folded-domain restraint, the H1-ProTalpha ff14sb condition was repeated with and without the preparatory Q-restrained equilibration stage. Omitting the restraint increased the analyzed H1 Domain1 RMSD by 0.016 nm, with a 95% confidence interval of 0.006-0.027 nm and $p = 0.00235$ in the linear mixed model described in SI Section S8. This control supports the use of Q restraints as an equilibration-stage safeguard for folded domains in dense condensates.

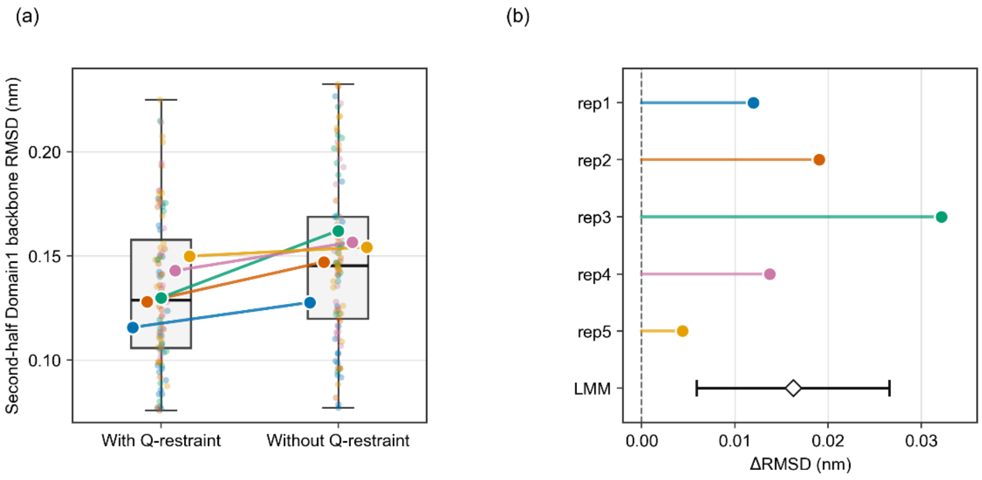

Figure S5. Native-contact Q restraints help preserve H1 Domain1 structure during folded-domain equilibration. The ff14sb H1-ProTalpha control compares simulations run with and without the preparatory Q-restrained equilibration stage. H1 Domain1 backbone RMSD was summarized as the per-domain mean for each H1 molecule in each independent repeat, using the same analysis window as Figure 3. Colors denote five independent repeats. (a) Per-domain RMSD distributions and paired repeat-level means for with-restraint and without-restraint workflows. (b) Paired increase in repeat-level RMSD after omitting the restraint, defined as $\Delta\text{RMSD} = \text{RMSD}_{\text{without Q}} - \text{RMSD}_{\text{with Q}}$. Positive

values indicate higher RMSD without the restraint. The linear mixed-model estimate was $\Delta\mathrm{RMSD} = 0.016$ nm, with a 95% confidence interval of 0.006-0.027 nm and $p = 0.00235$.

S7. Structural quality metrics

Structural quality was assessed using three metrics: steric clash score, ring penetration score, and chirality score.[27] These metrics were calculated with a lightweight geometry-check workflow based on MDAnalysis, SciPy cKDTree, and custom analyses. The workflow focused on structural pathologies that would prevent stable all-atom MD simulations.

S7.1 Steric clash score

A steric clash was counted when two non-hydrogen atoms were separated by less than 1.2 Å. Intra-residue atom pairs were excluded. For immediately adjacent residues, backbone-backbone and backbone-sidechain pairs were also excluded to avoid counting local bonded geometry. The steric clash score was reported as the percentage of residues containing at least one clashing heavy atom.

S7.2 Ring penetration score

Ring penetration was evaluated for PHE, TYR, TRP, HIS, and PRO rings. For each ring, the ring center and plane normal were determined by singular-value decomposition. Candidate penetrating covalent bonds were identified by spatial neighbor search. A bond was counted as penetrating a ring if the bond segment intersected the ring plane and the intersection point lay inside the ring polygon, as determined by a winding-number/accumulated-angle criterion. The score was reported as the percentage of ring-containing residues with at least one penetration.

S7.3 Chirality score

Chirality was evaluated at Cα atoms for standard chiral amino acids and at Cβ for ILE and THR. For each center, the signed volume defined by the scalar triple product of substituent vectors was compared with the expected handedness. A center with inverted signed volume was counted as a chirality violation. The score was reported as the percentage of evaluated chiral centers with incorrect handedness.

Table S4. Source-data summary of structural artifact metrics before and after CondenSimAdapter minimization. Values are mean $\pm$ SD across five independent repeats. All entries are percentages.

System or condition	Backmap clash	Minimized clash	Backmap ring	Minimized ring
CALVADOS	1.94 ± 0.14	0	0.44 ± 0.12	0
COCOMO2	2.31 ± 0.32	0	0.66 ± 0.15	0
HPS-Urry	2.23 ± 0.47	0	0.56 ± 0.13	0
Mpipi-Recharged	2.02 ± 0.23	0	0.35 ± 0.16	0
H1-ProTalpha	1.08 ± 0.25	0	0.22 ± 0.16	0
A1 LCD	2.30 ± 0.18	0	0.72 ± 0.12	0
DDX4 LCD	2.11 ± 0.37	0	0.58 ± 0.16	0
(GRGDSPYS)25	2.50 ± 0.29	0	0.51 ± 0.12	0
LAF1 RGG	2.65 ± 0.24	0	0.89 ± 0.28	0
TDP43 LCD	1.17 ± 0.16	0	0.50 ± 0.18	0
FUS	3.29 ± 1.43	0	0.83 ± 0.77	0
TDP43	7.05 ± 3.19	0	3.07 ± 2.21	0.01 ± 0.03
hnRNPA1S	6.20 ± 3.92	0	1.99 ± 2.00	0

S8. Statistical analysis and independent repeats

Artifact metrics in Figure 2 were summarized over independent CG snapshots and complete CG-to-AA construction repeats. Because these measurements are single-structure

outputs rather than trajectory time series, values are reported as mean $\pm$ SD over five repeats without block averaging.

For trajectory observables, per-domain time averages were used as sampling units. In Figure 3, each H1 Domain1 RMSD time series was averaged over the analyzed trajectory window for each H1 molecule in each independent repeat. This produced 100 values per force field: 5 repeats $\times$ 20 H1 domains. These per-domain mean RMSD values form the sampling unit shown in the violin plots.

The H1 RMSD reference structure was the initial folded H1 Domain1 structure used to define the MDP input. Backbone atoms (N, Cα, and C) in residues 22-96 were used for RMSD calculation after alignment to the reference domain.

For the Q-restraint control, we used a linear mixed model[28]:

$$\mathrm{RMSD}_{\mathrm{mean}} \sim \mathrm{Condition} + (1 \mid \mathrm{Replica}) + (1 \mid \mathrm{Monomer}).$$

The response variable was the per-domain mean RMSD over the final 5 ns of each 10 ns production trajectory, matching the Figure 3 analysis. The fixed effect was the presence or absence of the preparatory Q-restrained equilibration stage. Replica and monomer identity were treated as random effects. Satterthwaite degrees of freedom were used for p values through `lmerTest`. Omitting the restraint increased RMSD by 0.016 nm ($p = 2.35 \times 10^{-3}$; Figure S5).

S9. Energy stability after construction

To assess the short-timescale stability of the generated structures, each constructed system was simulated for 10 ns under NPT conditions using a 2 fs timestep. The relative potential energy was defined as:

$$\Delta E(t) = E(t) - E(0).$$

Values were summarized as mean SEM across five independent repeats. The energy profiles served as numerical stability diagnostics rather than as thermodynamic observables. Across all tested systems (including FUS LC with different CG force fields, H1-ProTalpha with various AA force fields, and diverse IDP/MDP condensates), no systematic energy divergence or integration instability was observed after the **CondenSimAdapter** minimization protocol.

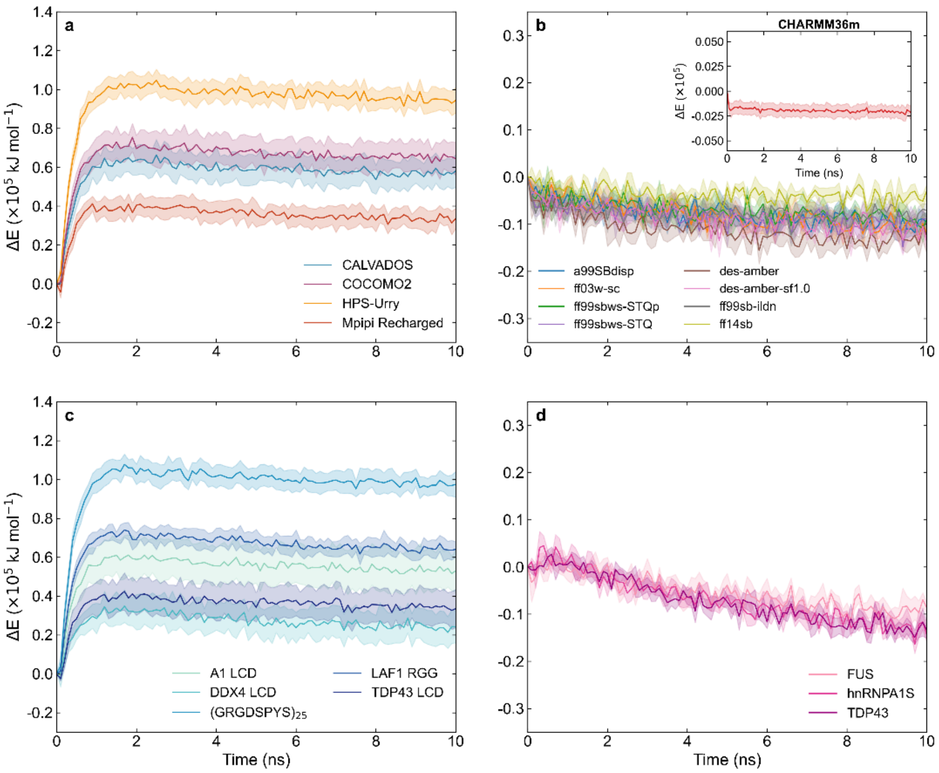

Figure S6. Potential energy evolution of biomolecular condensates after **CondenSimAdapter** construction. Relative potential energy, $\Delta E(t) = E(t) - E(0)$, is shown during 10 ns NPT production tests for (a) FUS LC constructed from four CG force fields, (b) H1-ProTalpha constructed with nine AA force fields, (c) IDP condensates, and (d) MDP condensates. Values are mean $\pm$ SEM over five independent repeats. The absence of systematic divergence indicates stable initial AA dynamics after staged relaxation, solvation, and solvated-system minimization.

S10. FUS LC 2 μs atomistic validation setup and analysis details

The 2 μs FUS LC system contained 40 chains, each with 163 residues. The CG condensate was generated with Mpipi-Recharged and converted to AA coordinates through the standard **CondenSimAdapter** workflow. The AA force field was amber99sbws-STQp,[17] and the water model was TIP4P/2005.[14] The system was solvated in an approximately 10 $\times$ 10 $\times$ 40 nm box and ionized with 0.15 M NaCl plus neutralizing counterions, yielding approximately 483,000 atoms after solvation and ionization.

The solvated system was minimized using the steepest-descent and conjugate-gradient protocol described in SI Section S5, followed by NPT equilibration until the volume stabilized. The production simulation was then run for 2 μs in the NVT ensemble at 300 K. Temperature was controlled with Langevin dynamics using a friction coefficient of 1.0 ps^{-1} and a 4 fs timestep. Hydrogen-bond constraints were applied, electrostatics were treated with PME, and nonbonded interactions used a 0.9 nm real-space cutoff. Coordinates were saved every 1 ns for the full system and every 0.1 ns for the protein coordinates used in structural analyses.

To quantify the statistical uncertainty of the radius-of-gyration autocorrelation analysis, we performed a chain-level bootstrap analysis using the 40 FUS LC chains in the 2 μs atomistic trajectory. For each bootstrap replicate, 40 chains were sampled with replacement, the mean R_g autocorrelation function was recalculated, and the relaxation time τ was obtained using the same fitting procedure as in the main analysis. A total of 2000 bootstrap replicates were used to estimate the 95% confidence band of the autocorrelation function and the uncertainty of the fitted relaxation time. This analysis yielded τ = 181 ns, with a bootstrap SD of 14.7 ns and a 95% confidence interval of 154-211 ns (Fig. S7), indicating that the ACF-derived relaxation time is robust to resampling of the 40-chain ensemble.

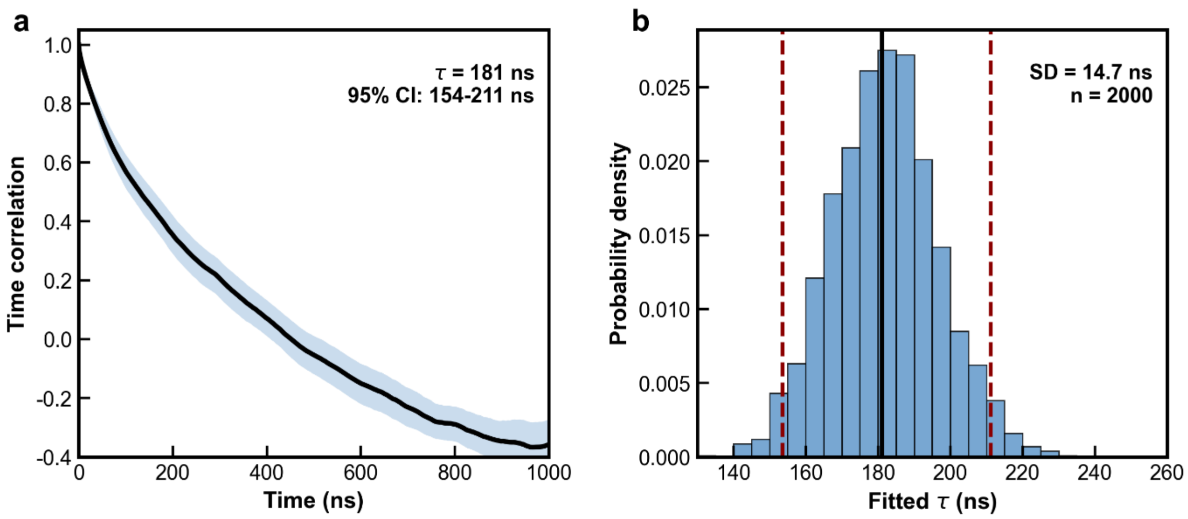

Figure S7 Mean R_g autocorrelation function from the 2 μs atomistic FUS LC trajectory, with the 95% chain-bootstrap confidence band shown by blue shading. Bootstrap samples were generated by resampling the 40 chains with replacement. (b) Distribution of fitted relaxation times from 2000 bootstrap replicates. The black vertical line marks the relaxation time fitted from the original 40-chain mean autocorrelation function, and the red dashed lines mark the 95% bootstrap confidence interval. The bootstrap analysis gives τ = 181 ns, bootstrap SD = 14.7 ns, and 95% confidence interval = 154-211 ns.

Diffusion coefficients were estimated using the propagator method described in the Zheng 2020.[29] The slab-normal (z) center-of-mass displacement distribution at lag time t was fitted to:

$$P(\Delta\xi, t) = A(4\pi Dt)^{-1/2}\exp[-\Delta\xi^2/(4Dt)].$$

The slab-normal diffusion coefficient reached approximately 0.45 $\mu\mathrm{m}^2\ \mathrm{s}^{-1}$ in the long-lag plateau region, close to the 0.404 $\mu\mathrm{m}^2\ \mathrm{s}^{-1}$ value reported by Zheng et al.

S11. Entanglement detection and Z1+ benchmark

CondenSimAdapter includes an optional entanglement check that is enabled by default. The check is intended to identify CG condensate configurations with elevated chain entanglement before AA reconstruction, which is particularly relevant for dense systems and for proteins that form solid-like or gel-like condensates. The analysis is based on a Z-code primitive path analysis (PPA) algorithm.[30] Each chain is iteratively contracted by node removal; an internal node is removed only when the removal triangle does not intersect bonds from other chains. The resulting primitive path is used to estimate the mean entanglement number per chain.

In the workflow, the built-in detector is invoked after CG structure generation. **CondenSimAdapter** can also call a locally installed Z1+[31] executable for higher-precision analysis when the user has obtained the required license and compiled Z1+ in the local environment.

CondenSimAdapter reports the mean entanglement number Z using four operational categories. Systems with $Z \leq 0.5$ are labeled OK and are treated as having no substantial entanglement. Systems with $0.5 < Z \leq 4.0$ are labeled NORMAL, corresponding to the typical range observed for condensate configurations that can proceed without intervention. Systems with $4.0 < Z \leq 7.0$ are labeled ELEVATED and should be inspected if such entanglement is unexpected for the target system. Systems with $Z > 7.0$ are labeled HIGH; in this regime, the recommended action is to regenerate or resample the CG condensate, for example by lowering the initial density. These thresholds are intended as empirical, workflow-specific quality-control guidelines rather than universal physical boundaries, and should therefore be interpreted relative to the protein system and condensate state under study.

In the condensate construction tests performed in this work, most generated configurations had mean entanglement number $\langle Z \rangle$ at or below 2 and therefore fell within the NORMAL range, or lower. Elevated values were interpreted as configuration-level warnings rather than automatic failures, because some proteins can form solid or gel phases in which increased topological complexity may be expected.

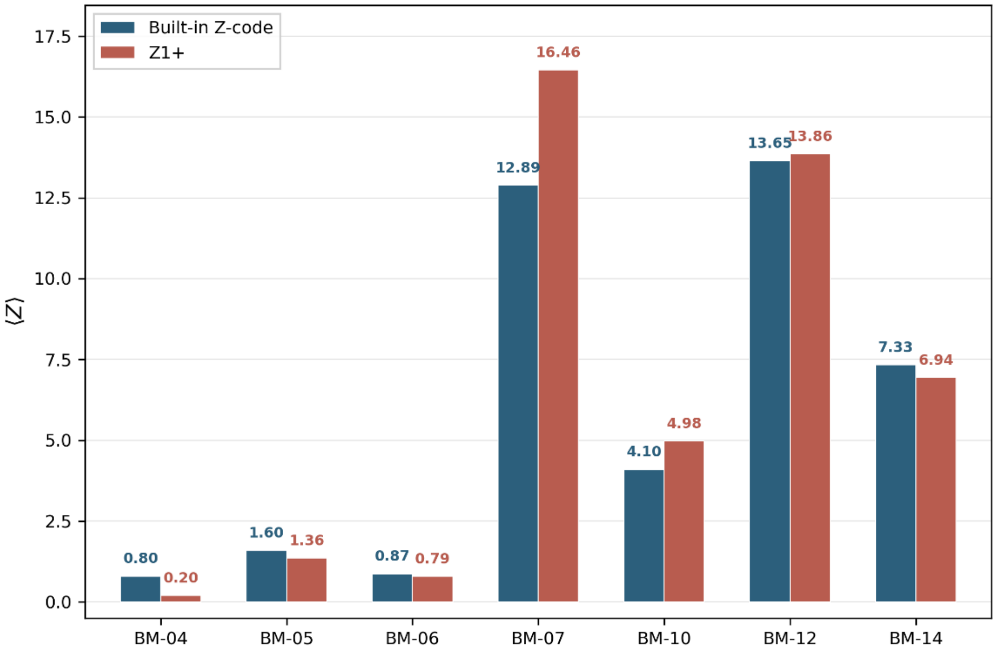

Figure S8. Comparison of mean entanglement number between the built-in Z-code detector and Z1+. The benchmark compares the **CondenSimAdapter** built-in primitive-path detector with the external Z1+ reference implementation across seven systems spanning low-entanglement and high-entanglement regimes. Each point reports the mean entanglement number for one benchmark system. The built-in detector reproduces the qualitative system ranking and gives high binary agreement with Z1+, supporting its use as a construction-stage warning metric.

Elevated entanglement is handled at the CG-configuration level rather than by post-processing the AA structure. Once a dense configuration has been converted to an AA representation, removing chain crossings would require large coordinated chain displacements and could introduce nonphysical structural rearrangements. For this reason, the practical strategy is to flag elevated entanglement before AA setup and regenerate or resample the CG condensate, for example by using a different seed or a lower initial density.

S12. Comparison with related workflows

CondenSimAdapter is designed for a specific stage of multiscale condensate simulation: converting pre-equilibrated CG condensate configurations into solvated AA systems for production MD. This scope differs from several related tools.

CHARMM-GUI Multicomponent Assembler (MCA) is a mature builder for AA multicomponent systems within the CHARMM ecosystem.[32] It is well suited for assembling proteins, nucleic acids, polymers, lipids, and other components directly at atomistic resolution. MCA and **CondenSimAdapter** therefore address different entry points into AA simulation: direct AA assembly in MCA and CG-to-AA conversion of pre-equilibrated dense protein condensates in **CondenSimAdapter**.

IPAMD is a CG-level platform for IDP condensate formation dynamics and large-scale sequence exploration.[33] It provides plugins and batch-processing capabilities for CG condensate simulation and analysis, making it especially useful for CG-stage condensate formation and screening.

OpenABC provides a flexible Python interface for CG biomolecular condensate simulations and includes a REMO-based CG-to-AA reconstruction utility.[34,35] It is valuable for CG model setup and can generate atomistic PDB structures. **CondenSimAdapter** builds on the same broad multiscale need but emphasizes dense-phase relaxation, solvation, topology generation, and systematic AA force-field integration after CG-to-AA conversion.

Table S5. Comparison of CondenSimAdapter with related condensate-modeling workflows. The table summarizes the main scope, starting point, force-field coverage, reconstruction strategy, and production-system preparation supported by each tool.

Feature	CHARMM-GUI MCA	IPAMD	OpenABC	CondenSimAdapter
Primary scope	AA multicomponent assembly	CG condensate dynamics	CG setup plus basic AA reconstruction	CG-to-production-AA condensate workflow
Starts from CG condensate trajectory	No	CG only	Yes, partly	Yes
CG force fields	Outside primary scope	HPS, HPS-T, CALVADOS variants, Mpipi	MOFF, HPS, Mpipi, SMOG	CALVADOS, COCOMO2, HPS-Urry, Mpipi-Recharged
Backmapping	Outside primary scope	CG-stage only	REMO	cg2all by default; modular
Dense-phase clash rescue	Standard AA preparation	CG-stage only	Downstream integration	Gaussian + soft-core + standard relaxation
AA force fields	CHARMM ecosystem	CG-stage only	Downstream integration	nine registered force fields
Solvation/topology	Automated for AA assembly	CG-stage only	Downstream integration	automated
Folded-domain handling	AA model dependent	CG elastic/rigid treatments	CG native memory terms	AA Q restraints through PLUMED
Nucleic-acid support	Yes	Not yet	DNA/RNA CG support	future extension
Main output	AA simulation system	CG trajectories/analysis	CG trajectories and reconstructed AA PDBs	solvated AA systems for production MD

Together, these tools cover different stages of condensate modeling. IPAMD and OpenABC can generate or explore CG condensate configurations, MCA can assemble AA multicomponent systems directly, and **CondenSimAdapter** fills the CG-to-production-AA conversion stage for dense protein condensates.

S13. Supplementary tables

Table S1 Amino acid sequences of IDPs investigated in this work.

System	Sequence
FUS LC	MASND YTQQA TQSYG AYPTQ PGQGY SQQSS QPYGQ QSYSG YSQST DTSGY
	GQSSY SSYGQ SQNTG YGTQS TPQGY GSTGG YGSSQ SSQSS YGQQS SYPGY
	GQQPA PSSTS GSYGS SSQSS SYGQP QSGSY SQQPS YGGQQ QSYGQ QQSYN
	PPQGY GQQNQ YNS
ProTα	GPMSD AAVDT SSEIT TKDLK EKKEV VEEAE NGRDA PANGN ANEEN GEQEA
	DNEVD EEEEE GGEEE EEEEE GDGEE EDGDE DEEAE SATGK RAAED DEDDD
	VDTKK QKTDE DD
LAF1 RGG	MESNQ SNNGG SGNAA LNRGG RYVPP HLRGG DGGAA AAASA GGDDR RGGAG
	GGGYR RGGGN SGGGG GGGYD RGYND NRDDR DNRGG SGGYG RDRNY EDRGY
	NGGGG GGGNR GYNNN RGGGG GGYNR QDRGD GGSSN FSRGG YNNRD EGSDN
	RGSGR SYNND RRDNG GDG
TDP43 LCD	NRQLE RSGRF GGNPG GFGNQ GGFGN SRGGG AGLGN NQGSN MGGGM NFGAF
	SINPA MMAAA QAALQ SSWGM MGMLA SQQNQ SGPSG NNQNQ GNMQR EPNQA
	FGSGN NSYSG SNSGA AIGWG SASNA GSGSG FNGGF GSSMD SKSSG WGM
DDX4 LCD	MGDED WEAEI NPHMS SYVPI FEKDR YSGEN GDNFN RTPAS SSEMD DGPSR
	RDHFM KSGFA SGRNF GNRDA GECNK RDNTS TMGGF GVGKS FGNRG FSNSR
	FEDGD SSGFW RESSN DCEDN PTRNR GFSKR GGYRD GNNSE ASGPY RRGGR
	GSFRG CRGGF GLGSP NNDLD PDECM QRTGG LFGSR RPVLS GTGNG DTSQS
	RSGSG SERGG YKGLN EEVIT GSGKN SWKSE AEGGE S
A1 LCD	MASAS SSQRG RSGSG NFGGG RGGGF GGNDN FGRGG NFSGR GGFGG SRGGG
	GYGGS GDGYN GFGND GSNFG GGGSY NDFGN YNNQS SNFGP MKGGN FGGRS
	SGPYG GGGQY FAKPR NQGGY GGSSS SSSYG SGRRF
$(GRGDSPYS)_{25}$	GRGDS PYSGR GDSPY SGRGD SPYSG RGDSP YSGRG DSPYS GRGDS PYSGR
	GDSPY SGRGD SPYSG RGDSP YSGRG DSPYS GRGDS PYSGR GDSPY SGRGD
	SPYSG RGDSP YSGRG DSPYS GRGDS PYSGR GDSPY SGRGD SPYSG RGDSP
	YSGRG DSPYS GRGDS PYSGR GDSPY SGRGD SPYSG RGDSP YSGRG DSPYS

Table S2 Amino acid sequences of the MDPs investigated in this work. Structured domains are highlighted in red.

System	Sequence
H1	TENST SAPAA KPKRA KASKK STDHP KYSDM IVAAI QAEKN RAGSS RQSIQ KYIKS HYKVG ENADS QIKLS IKRLV TTGVL KQTKG VGASG SFRLA KSDEP KKSVA FKKTK KEIKK VATPK KASKP KKAAS KAPTK KPKAT PVKKA KKKLA ATPKK AKKPK TVKAK PVKAS KPKKA KPVKP KAKSS AKRAG KKK
TDP43	MSEYI RVTED ENDEP IEIPS EDDGT VLLST VTAQF PGACG LRYRN PVSQC MRGVR LVEGI LHAPD AGWGN LVYVV NYPKD NKRKM DETDA SSAVK VKRAV QKTSD LIVLG LPWKT TEQDL KEYFS TFGEV LMVQV KKDLK TGHSK GFGFV RFTEY ETQVK VMSQR HMIDG RWCDC KLPNS KQSQD EPLRS RKVFV GRCTE DMTED ELREF FSQYG DVMDV FIPKP FRAFA FVTFA DDQIA QSLCG EDLII KGISV HISNA EPKHN SNRQL ERSGR FGGNP GGFGN QGGFG NSRGG GAGLG NNQGS NMGGG MNFGA FSINP AMMAA AQAAL QSSWG MMGML ASQQN QSGPS GNNQN QGNMQ REPNQ AFGSG NNSYS GSNSG AAIGW GSASN AGSGS GFNGG FGSSM DSKSS GWGM
HnRNPA1S	MSKSE SPKEP EQLRK LFIGG LSFET TDESL RSHFE QWGTL TDCVV MRDPN TKRSR GFGFV TYATV EEVDA AMNAR PHKVD GRVVE PKRAV SREDS QRPGA HLTVK KIFVG GIKED TEEHH LRDYF EQYGK IEVIE IMTDR GSGKK RGFAF VTFDD HDSVD KIVIQ KYHTV NGHNC EVRKA LSKQE MASAS SSQRG RSGSG NFGGG RGGGF GGNDN FGRGG NFSGR GGFGG SRGGG GYGGS GDGYN GFGND GSNFG GGGNY NNQSS NFGPM KGGNF GGRSS GPYGG GGQYF AKPRN QGGYG GSSSS SSYGS GRRF
FUS	MASND YTQQA TQSYG AYPTQ PGQGY SQQSS QPYGQ QSYSG YSQST DTSGY GQSSY SSYGQ SQNTG YGTQS TPQGY GSTGG YGSSQ SSQSS YGQQS SYPGY GQQPA PSSTS GSYGS SSQSS SYGQP QSGSY SQQPS YGGQQ QSYGQ QQSYN PPQGY GQQNQ YNSSS GGGGG GGGGG NYGQD QSSMS SGGGS GGGYG NQDQS GGGGS GGYGQ QDRGG RGRGG SGGGG GGGGG GYNRS SGGYE PRGRG GGRGG RGGMG GSDRG GFNKF GGPRD QGSRH DSEQD NSDNN TIFVQ GLGEN VTIES VADYF KQIGI IKTNK KTGQP MINLY TDRET GKLKG EATVS FDDPP SAKAA IDWFD GKEFS GNPIK VSFAT RRADF NRGGG NGRGG RGRGG PMGRG GYGGG GSGGG GRGGF PSGGG GGGGQ QRAGD WKCPN PTCEN MNFSW RNECN QCKAP KPDGP GGGPG GSHMG GNYGD DRRGG RGGYD RGGYR GRGGD RGGFR GGRGG GDRGG FGPGK MDSRG EHRQD RRERP Y

Table S3. Summary of energy minimization efficiency and final maximum forces for tested systems. The "steps used" represent the cumulative iterations from both the steepest descent (SD) and conjugate gradient (CG) minimization.

System	CG force field	All-atom force field	Step used	F_{max} $(\mathrm{kJ} \cdot \mathrm{mol}^{-1} \cdot \mathrm{nm}^{-1})$
FUS LC	CALVADOS	ff99sb-STQp	1395	438
	COCOMO2		959	464
	Mpipi-Recharged		1535	310
	HPS-Urry		4295	328
H1-ProTalpha	Mpipi-Recharged	a99SBdisp	335	478
		ff03w-sc	3869	474
		ff99sbws-STQp	2985	328
		ff99sbws-STQ	3987	492
		des-amber	4011	449
		des-amber-sf1.0	2664	381
		ff99sb-ildn	3763	466
		ff14sb	4176	424
		charmm36m	791	398
LAF1 RGG	CALVADOS	ff99sbws-STQp	1404	448
TDP43 LCD			4692	410
DDX4 LCD			2060	368
$(\mathrm{GRGDSPYS})_{25}$			5001	344
A1 LCD			1296	489
hnRNPA1S			1014	485
TDP43			674	458
FUS			2070	445

Table S6. Simplified names used in the main text, figures, and tables.

Simplified name	Full name or description
FUS LC	low-complexity domain of fused in sarcoma (FUS)
H1-ProTalpha	linker histone H1 and prothymosin alpha condensate
LAF1 RGG	RGG/RG-rich domain of the LAF-1 DEAD-box RNA helicase
TDP43 LCD	low-complexity domain of TAR DNA-binding protein 43 (TDP43)
DDX4 LCD	low-complexity domain of DEAD-box helicase 4 (DDX4)
$(GRGDSPYS)_{25}$	25-repeat synthetic peptide with the repeat sequence GRGDSPYS
A1 LCD	low-complexity domain of heterogeneous nuclear ribonucleoprotein A1 (hnRNPA1)
FUS	full-length fused in sarcoma
TDP43	full-length TAR DNA-binding protein 43
hnRNPA1S	short isoform of heterogeneous nuclear ribonucleoprotein A1

Reference

(1) Abraham, M. J.; Murtola, T.; Schulz, R.; Páll, S.; Smith, J. C.; Hess, B.; Lindahl, E. GROMACS: High Performance Molecular Simulations through Multi-Level Parallelism from Laptops to Supercomputers. *SoftwareX* **2015**, *1–2*, 19–25. https://doi.org/10.1016/j.softx.2015.06.001.

(2) Eastman, P.; Galvelis, R.; Peláez, R. P.; Abreu, C. R. A.; Farr, S. E.; Gallicchio, E.; Gorenko, A.; Henry, M. M.; Hu, F.; Huang, J.; Krämer, A.; Michel, J.; Mitchell, J. A.; Pande, V. S.; Rodrigues, J. P.; Rodriguez-Guerra, J.; Simmonett, A. C.; Singh, S.; Swails, J.; Turner, P.; Wang, Y.; Zhang, I.; Chodera, J. D.; De Fabritiis, G.; Markland, T. E.

OpenMM 8: Molecular Dynamics Simulation with Machine Learning Potentials. *J. Phys. Chem. B* **2024**, *128* (1), 109–116. https://doi.org/10.1021/acs.jpcb.3c06662.

(3) Case, D. A.; Cerutti, D. S.; Cruzeiro, V. W. D.; Darden, T. A.; Duke, R. E.; Ghazimirsaeed, M.; Giambaşu, G. M.; Giese, T. J.; Götz, A. W.; Harris, J. A.; Kasavajhala, K.; Lee, T.-S.; Li, Z.; Lin, C.; Liu, J.; Miao, Y.; Salomon-Ferrrer, R.; Shen, J.; Snyder, R.; Swails, J.; Walker, R. C.; Wang, J.; Wu, X.; Zeng, J.; Cheatham III, T. E.; Roe, D. R.; Roitberg, A.; Simmerling, C.; York, D. M.; Nagan, M. C.; Merz, K. M. Jr. Recent Developments in Amber Biomolecular Simulations. *J. Chem. Inf. Model.* **2025**, *65* (15), 7835–7843. https://doi.org/10.1021/acs.jcim.5c01063.

(4) Tesei, G.; Lindorff-Larsen, K. Improved Predictions of Phase Behaviour of Intrinsically Disordered Proteins by Tuning the Interaction Range. *Open Res. Eur.* **2023**, *2*, 94. https://doi.org/10.12688/openreseurope.14967.2.

(5) Cao, F.; von Bülow, S.; Tesei, G.; Lindorff-Larsen, K. A Coarse-grained Model for Disordered and Multi-domain Proteins. *Protein Sci.* **2024**, *33* (11). https://doi.org/10.1002/pro.5172.

(6) Jussupow, A.; Bartley, D.; Lapidus, L. J.; Feig, M. COCOMO2: A Coarse-Grained Model for Interacting Folded and Disordered Proteins. *J. Chem. Theory Comput.* **2025**, *21* (4), 2095–2107. https://doi.org/10.1021/acs.jctc.4c01460.

(7) R. Tejedor, A.; Aguirre Gonzalez, A.; Maristany, M. J.; Chew, P. Y.; Russell, K.; Ramirez, J.; Espinosa, J. R.; Collepardo-Guevara, R. Chemically Informed Coarse-Graining

of Electrostatic Forces in Charge-Rich Biomolecular Condensates. *ACS Cent. Sci.* **2025**, *11* (2), 302–321. https://doi.org/10.1021/acscentsci.4c01617.

(8) Regy, R. M.; Thompson, J.; Kim, Y. C.; Mittal, J. Improved Coarse-grained Model for Studying Sequence Dependent Phase Separation of Disordered Proteins. *Protein Sci.* **2021**, *30* (7), 1371–1379. https://doi.org/10.1002/pro.4094.

(9) Anderson, J. A.; Glaser, J.; Glotzer, S. C. HOOMD-Blue: A Python Package for High-Performance Molecular Dynamics and Hard Particle Monte Carlo Simulations. *Comput. Mater. Sci.* **2020**, *173*, 109363. https://doi.org/10.1016/j.commatsci.2019.109363.

(10) Heo, L.; Feig, M. One Bead per Residue Can Describe All-Atom Protein Structures. *Structure* **2024**, *32* (1), 97-111.e6. https://doi.org/10.1016/j.str.2023.10.013.

(11) Onufriev, A.; Bashford, D.; Case, D. A. Exploring Protein Native States and Large-scale Conformational Changes with a Modified Generalized Born Model. Funct. Bioinforma. 2004, 55 (2), 383–394. https://doi.org/10.1002/prot.20033.

(12) Li, Y.; Nam, K. Repulsive Soft-Core Potentials for Efficient Alchemical Free Energy Calculations. *J. Chem. Theory Comput.* **2020**, *16* (8), 4776–4789. https://doi.org/10.1021/acs.jctc.0c00163.

(13) Gapsys, V.; Seeliger, D.; de Groot, B. L. New Soft-Core Potential Function for Molecular Dynamics Based Alchemical Free Energy Calculations. *J. Chem. Theory Comput.* **2012**, *8* (7), 2373–2382. https://doi.org/10.1021/ct300220p.

(14) Abascal, J. L. F.; Vega, C. A General Purpose Model for the Condensed Phases of Water: TIP4P/2005. *J. Chem. Phys.* **2005**, *123* (23), 234505. https://doi.org/10.1063/1.2121687.

(15) Essmann, U.; Perera, L.; Berkowitz, M. L.; Darden, T.; Lee, H.; Pedersen, L. G. A Smooth Particle Mesh Ewald Method. *J. Chem. Phys.* **1995**, *103* (19), 8577–8593. https://doi.org/10.1063/1.470117.

(16) Robustelli, P.; Piana, S.; Shaw, D. E. Developing a Molecular Dynamics Force Field for Both Folded and Disordered Protein States. *Proc. Natl. Acad. Sci.* **2018**, *115* (21). https://doi.org/10.1073/pnas.1800690115.

(17) Phan, T. M.; Mohanty, P.; Mittal, J. Optimized Protein-Water Interactions and Torsional Refinements Yield Balanced Atomistic Protein Force Fields. *Nat. Commun.* **2025**, *16* (1). https://doi.org/10.1038/s41467-025-65603-4.

(18) Tang, W. S.; Fawzi, N. L.; Mittal, J. Refining All-Atom Protein Force Fields for Polar-Rich, Prion-like, Low-Complexity Intrinsically Disordered Proteins. *J. Phys. Chem. B* **2020**, *124* (43), 9505–9512. https://doi.org/10.1021/acs.jpcb.0c07545.

(19) Piana, S.; Robustelli, P.; Tan, D.; Chen, S.; Shaw, D. E. Development of a Force Field for the Simulation of Single-Chain Proteins and Protein–Protein Complexes. *J. Chem. Theory Comput.* **2020**, *16* (4), 2494–2507. https://doi.org/10.1021/acs.jctc.9b00251.

(20) Lindorff-Larsen, K.; Piana, S.; Palmo, K.; Maragakis, P.; Klepeis, J. L.; Dror, R. O.; Shaw, D. E. Improved Side-Chain Torsion Potentials for the Amber ff99SB Protein Force

Field. *Proteins Struct. Funct. Bioinforma.* **2010**, *78* (8), 1950–1958. https://doi.org/10.1002/prot.22711.

(21) Maier, J. A.; Martinez, C.; Kasavajhala, K.; Wickstrom, L.; Hauser, K. E.; Simmerling, C. ff14SB: Improving the Accuracy of Protein Side Chain and Backbone Parameters from ff99SB. *J. Chem. Theory Comput.* **2015**, *11* (8), 3696–3713. https://doi.org/10.1021/acs.jctc.5b00255.

(22) Huang, J.; Rauscher, S.; Nawrocki, G.; Ran, T.; Feig, M.; de Groot, B. L.; Grubmüller, H.; MacKerell, A. D. CHARMM36m: An Improved Force Field for Folded and Intrinsically Disordered Proteins. *Nat. Methods* **2017**, *14* (1), 71–73. https://doi.org/10.1038/nmeth.4067.

(23) Bussi, G.; Donadio, D.; Parrinello, M. Canonical Sampling through Velocity Rescaling. *J. Chem. Phys.* **2007**, *126* (1). https://doi.org/10.1063/1.2408420.

(24) Bernetti, M.; Bussi, G. Pressure Control Using Stochastic Cell Rescaling. *J. Chem. Phys.* **2020**, *153* (11), 114107. https://doi.org/10.1063/5.0020514.

(25) Best, R. B.; Hummer, G.; Eaton, W. A. Native Contacts Determine Protein Folding Mechanisms in Atomistic Simulations. *Proc. Natl. Acad. Sci.* **2013**, *110* (44), 17874–17879. https://doi.org/10.1073/pnas.1311599110.

(26) Bonomi, M.; Bussi, G.; Camilloni, C.; Tribello, G. A.; Banáš, P.; Barducci, A.; Bernetti, M.; Bolhuis, P. G.; Bottaro, S.; Branduardi, D.; Capelli, R.; Carloni, P.; Ceriotti, M.; Cesari, A.; Chen, H.; Chen, W.; Colizzi, F.; De, S.; De La Pierre, M.; Donadio, D.; Drobot, V.; Ensing, B.; Ferguson, A. L.; Filizola, M.; Fraser, J. S.; Fu, H.; Gasparotto, P.;

Gervasio, F. L.; Giberti, F.; Gil-Ley, A.; Giorgino, T.; Heller, G. T.; Hocky, G. M.; Iannuzzi, M.; Invernizzi, M.; Jelfs, K. E.; Jussupow, A.; Kirilin, E.; Laio, A.; Limongelli, V.; Lindorff-Larsen, K.; Löhr, T.; Marinelli, F.; Martin-Samos, L.; Masetti, M.; Meyer, R.; Michaelides, A.; Molteni, C.; Morishita, T.; Nava, M.; Paissoni, C.; Papaleo, E.; Parrinello, M.; Pfaendtner, J.; Piaggi, P.; Piccini, G.; Pietropaolo, A.; Pietrucci, F.; Pipolo, S.; Provasi, D.; Quigley, D.; Raiteri, P.; Raniolo, S.; Rydzewski, J.; Salvalaglio, M.; Sosso, G. C.; Spiwok, V.; Šponer, J.; Swenson, D. W. H.; Tiwary, P.; Valsson, O.; Vendruscolo, M.; Voth, G. A.; White, A.; The PLUMED consortium. Promoting Transparency and Reproducibility in Enhanced Molecular Simulations. *Nat. Methods* **2019**, *16* (8), 670–673. https://doi.org/10.1038/s41592-019-0506-8.

(27) Ugarte La Torre, D.; Sugita, Y. CGBack: Diffusion Model for Backmapping Large-Scale and Complex Coarse-Grained Molecular Systems. *J. Chem. Inf. Model.* **2025**, *65* (19), 9974–9986. https://doi.org/10.1021/acs.jcim.5c01281.

(28) Kuznetsova, A.; Brockhoff, P. B.; Christensen, R. H. B. lmerTest Package: Tests in Linear Mixed Effects Models. *J. Stat. Softw.* **2017**, *82* (13), 1–26. https://doi.org/10.18637/jss.v082.i13.

(29) Zheng, W.; Dignon, G. L.; Jovic, N.; Xu, X.; Regy, R. M.; Fawzi, N. L.; Kim, Y. C.; Best, R. B.; Mittal, J. Molecular Details of Protein Condensates Probed by Microsecond Long Atomistic Simulations. *J. Phys. Chem. B* **2020**, *124* (51), 11671–11679. https://doi.org/10.1021/acs.jpcb.0c10489.

(30) Kröger, M. Shortest Multiple Disconnected Path for the Analysis of Entanglements in Two- and Three-Dimensional Polymeric Systems. *Comput. Phys. Commun.* **2005**, *168* (3), 209–232. https://doi.org/10.1016/j.cpc.2005.01.020.

(31) Kröger, M.; Dietz, J. D.; Hoy, R. S.; Luap, C. The Z1+ Package: Shortest Multiple Disconnected Path for the Analysis of Entanglements in Macromolecular Systems. *Comput. Phys. Commun.* **2023**, *283*, 108567. https://doi.org/10.1016/j.cpc.2022.108567.

(32) Kern, N. R.; Lee, J.; Choi, Y. K.; Im, W. CHARMM-GUI Multicomponent Assembler for Modeling and Simulation of Complex Multicomponent Systems. *Nat. Commun.* **2024**, *15* (1), 5459. https://doi.org/10.1038/s41467-024-49700-4.

(33) Liu, X.-Y.; Zhu, Y.-L.; Jiang, Y.-Z.; Shi, S.-K.; Zhao, L.; Lu, Z.-Y. IPAMD: A Plugin-Based Software for Biomolecular Condensate Simulations. *J. Chem. Theory Comput.* **2025**, *21* (11), 5746–5756. https://doi.org/10.1021/acs.jctc.5c00147.

(34) Liu, S.; Wang, C.; Latham, A. P.; Ding, X.; Zhang, B. OpenABC Enables Flexible, Simplified, and Efficient GPU Accelerated Simulations of Biomolecular Condensates. *PLOS Comput. Biol.* **2023**, *19* (9), e1011442. https://doi.org/10.1371/journal.pcbi.1011442.

(35) Li, Y.; Zhang, Y. REMO: A New Protocol to Refine Full Atomic Protein Models from C-Alpha Traces by Optimizing Hydrogen-Bonding Networks. *Proteins Struct. Funct. Bioinforma.* **2009**, *76* (3), 665–676. https://doi.org/10.1002/prot.22380.
